# Supplementary material for: Formation of Citrazinic Acid Ions and Their Contribution to Optical and Magnetic Features of Carbon Nanodots: A Combined Experimental and Computational Approach
Source: Materials (Basel). 2021 Feb 6;14(4):770. doi: 10.3390/ma14040770 (PMC7914458; doi:10.3390/ma14040770)

materials

MDPI

Supporting Information

for

Formation of citrazinic acid ions and their contribution to optical and magnetic features: a combined experimental and computational approach.

Francesca Mocci [1], Chiara Olla [2], Antonio Cappai [2], Riccardo Corpino [2], Pier Carlo Ricci [2], Daniele Chiriu [2], Marcello Salis [2], and Carlo Maria Carbonaro [2,*]

Content:

details on the calculation of solvation free energy and pKa

The starting data are the sum of electron and thermal energies (E_{TE}) for the molecules of a defined thermodynamic cycle in gaseous phases and with implicit solvent. Taking cycle 1 in Figure 1 as an example, ΔG_{solv} is the difference between the energy in solvent and the one in gaseous phase for each species.

$$\triangle G_{solv}(CZA) = E_{TE}(CZA_{solv}) - E_{TE}(CZA_{gas})$$

$$\triangle G_{solv}(CZA^{-}) = E_{TE}(CZA^{-}_{solv}) - E_{TE}(CZA^{-}_{gas})$$

ΔG_g is the difference between the two pairs of gas molecules on top of the cycle

$$\triangle G_g(CZA^{-}) = E_{TE}(CZA^{-}_{gas}) + E_{TE}(H_3O^{+}_{gas}) - E_{TE}(CZA_{gas}) - E_{TE}(H_2O_{gas})$$

Finally, ΔG_{sol} is the difference between the calculated terms including the experimental values of ΔG_g for H_2O and H_3O^+

$$\triangle G_{sol} = \triangle G_g(CZA^{-}) + \triangle G_s(CZA^{-}) + \triangle G_s(H_3O^{+}) - \triangle G_s(CZA) - \triangle G_s(H_2O)$$

From the value of ΔG_{sol} one can retrive the pK_a value

$$pK_a = \frac{\triangle G_{sol}}{1.364} - \log|H_2O| - c$$

where c is a correction value (Chemical Physics Letters 367, 145–149, 2003)

Figure S1: calculated absorption spectra for the CZA+CZA1MA (a) and CZA+CZA1M (b) equilibria

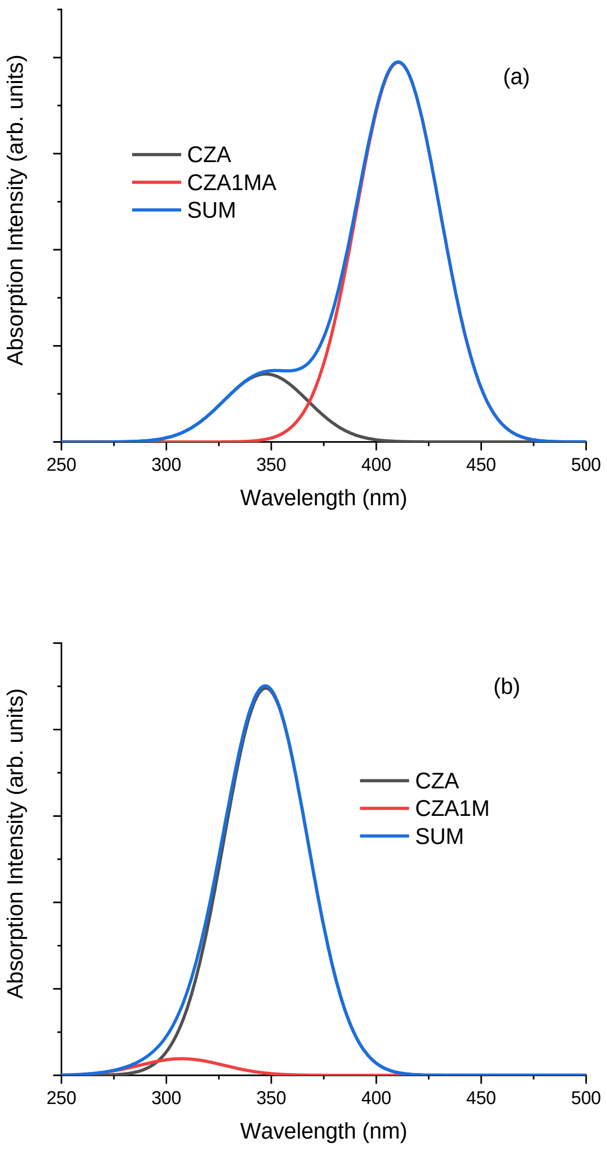

Table S1: GIAO isotropic Magnetic Shielding in water or dmso for CZA, its conjugated bases, its most stable dimer, and CZA+1 DMSO molecule.

GIAO isotropic Magnetic Shielding (ppm) for CZA calculated at the B3LYP/6311++G(d,p)/PCM(water)// B3LYP/6311++G(d,p)/PCM(water)

C 1	20,9605
C 2	15,2677
C 3	64,6028
C 4	35,0474
C 5	93,2965
H 6	25,1253
H 7	25,837
O 8	197,9442
H 9	26,3272
C 10	10,844
O 11	-73,6314
O 12	125,5298
H 13	25,2102
N 14	73,9744
H 15	23,7799
O16	-7,1832

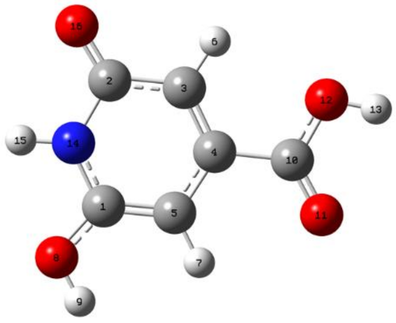

GIAO isotropic Magnetic Shielding (ppm) for CZA calculated at the B3LYP/6311++G(d,p)/PCM(dmso) //B3LYP/6311++G(d,p)/PCM(dmso)

C 1	20,997
C 2	15,3209
C 3	64,5291
C 4	35,0844
C 5	93,3851
H 6	25,1236
H 7	25,8441
O 8	198,0302
H 9	26,3448
C 10	10,858
O 11	-73,8662
O 12	125,5311
H 13	25,2209
N 14	74,0423
H 15	23,7886
O 16	-8,021

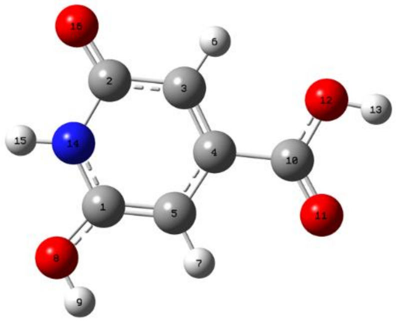

GIAO isotropic Magnetic Shielding (ppm) for CZA1M calculated at the B3LYP/6311++G(d,p)/PCM(dmso) //B3LYP/6311++G(d,p)/PCM(dmso)

C 1	22,8151
C 2	14,2285
C 3	69,1661
C 4	21,3045
C 5	91,767
H 6	25,3047
H 7	25,6895
O 8	203,6326
H 9	26,7276
C 10	10,161
O 11	7,1694
O 12	19,2876
N 13	78,568
H 14	24,1195
O 15	17,9207

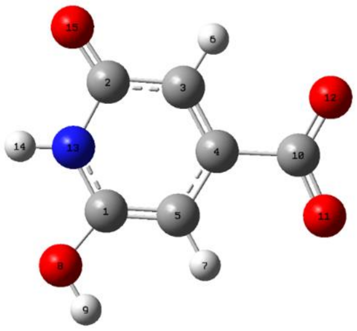

GIAO isotropic Magnetic Shielding (ppm) for CZA1MA calculated at the B3LYP/6311++G(d,p)/PCM(dmso) //B3LYP/6311++G(d,p)/PCM(dmso)		
C 1	11,8046	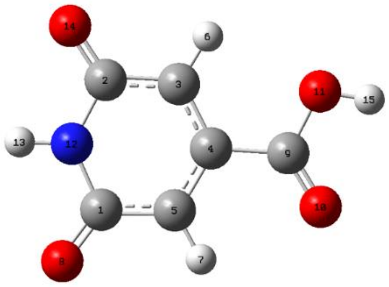
C 2	11,2729	
C 3	85,1613	
C 4	36,0027	
C 5	85,3718	
H 6	26,2033	
H 7	26,241	
O 8	40,1518	
C 9	7,332	
O 10	-58,5163	
O 11	122,9345	
N 12	55,5363	
H 13	24,4421	
O 14	37,9213	
H 15	25,6173	

GIAO isotropic Magnetic Shielding (ppm) for CZA head to head dimer calculated at the ωB97XD/6-311++G(d,p)/PCM(dmso) //B3LYP/6311++G(d,p)/PCM(dmso)		
O 1	16,2372	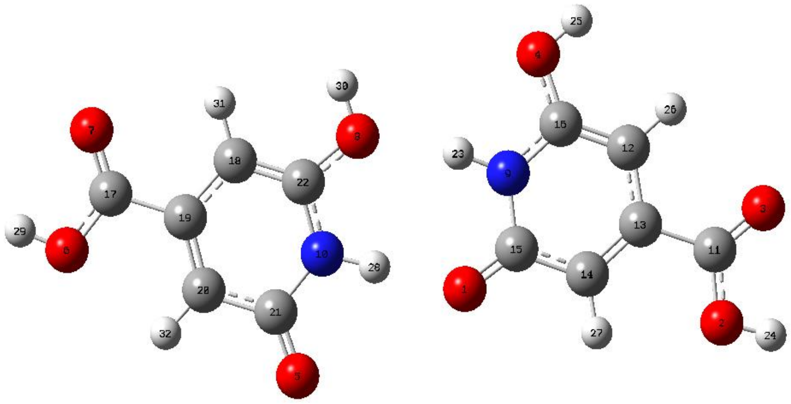
O 2	130,2828	
O 3	-66,111	
O 4	200,4352	
O 5	-4,1443	
O 6	130,9134	
O 7	-65,3002	
O 8	202,6725	
N 9	75,9163	
N 10	70,4327	
C 11	13,3488	
C 12	92,9917	
C 13	34,9026	
C 14	66,1651	
C 15	14,7734	
C 16	22,425	
C 17	12,5301	
C 18	95,4612	
C 19	36,7016	
C 20	63,8339	
C 21	15,2587	
C 22	22,3147	
H 23	21,2424	
H 24	25,3061	
H 25	26,4289	
H 26	25,7355	
H 27	25,034	
H 28	18,4729	
H 29	25,3749	
H 30	26,2763	
H 31	25,8519	
H 32	25,1208	

GIAO isotropic Magnetic Shielding (ppm) for CZA tail to tail dimer calculated at the ωB97XD/6-311++G(d,p)/PCM(dmso) //B3LYP/6311++G(d,p)/PCM(dmso)

O 1	200,8223
O 2	-28,6683
O 3	115,2515
O 4	-4,7398
O 5	-3,9212
O 6	115,1431
O 7	-28,9122
O 8	201,4582
N 9	76,5467
N 10	74,4777
C 11	22,4126
C 12	16,8298
C 13	65,0224
C 14	34,8483
C 15	95,4228
C 16	6,2634
C 17	16,7599
C 18	23,3393
C 19	93,5104
C 20	34,2529
C 21	66,2619
C 22	6,3085
H 23	27,0403
H 24	18,5218
H 25	25,0735
H 26	25,8261
H 27	26,4969
H 28	18,4751
H 29	23,8887
H 30	25,6894
H 31	25,1003
H 32	24,1521

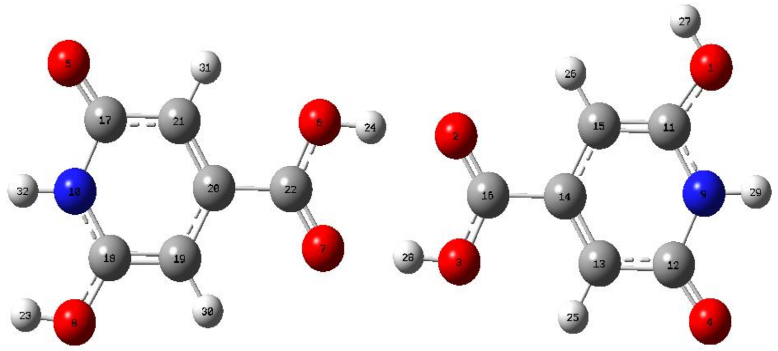

GIAO isotropic Magnetic Shielding (ppm) for CZA head to tail dimer calculated at the ωB97XD/6-311++G(d,p)/PCM(dmso) //B3LYP/6311++G(d,p)/PCM(dmso)

O 1	201,5907
O 2	-38,9228
O 3	108,8461
O 4	-1,5128
O 5	199,2388
O 6	-66,7959
O 7	130,2512
O 8	43,6046
N 9	77,2334
N 10	72,6617
C 11	22,603
C 12	16,8182
C 13	65,6382
C 14	33,4132
C 15	95,0634
C 16	7,5609
C 17	21,9188
C 18	13,5205
C 19	67,3309
C 20	34,5036
C 21	91,4607
C 22	12,9858
H 23	25,0876
H 24	25,7584

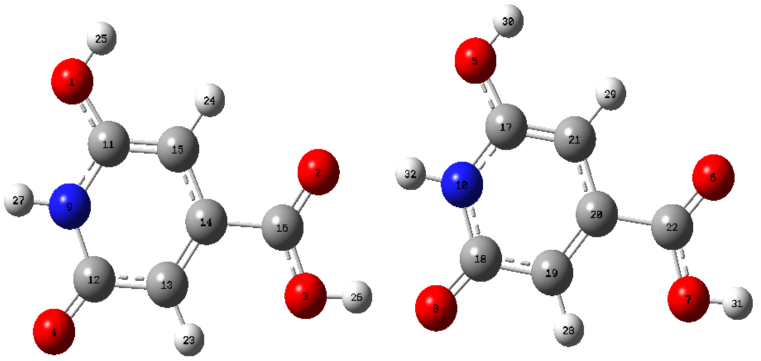

H 25	26,5322
H 26	16,6472
H 27	23,8962
H 28	25,0984
H 29	25,6239
H 30	26,3794
H 31	25,2817
H 32	18,7653

GIAO isotropic Magnetic Shielding (ppm) for CZA + DMSO system, with DMSO molecule near the carboxylic group, calculated at the ωB97XD/6311++G(d,p)/PCM(dmso)//B3LYP/6311++G(d,p)/PCM(dmso)

C 1	22,8587
C 2	95,3699
N 3	77,723
O 4	202,2792
C 5	32,7393
C 6	10,0447
O 7	-53,2107
O 8	109,9285
H 9	17,6384
H 10	26,5952
C 11	16,4198
H 12	23,9761
O 13	1,0371
C 14	66,6411
H 15	25,1925
H 16	25,8873
S 17	182,2015
C 18	142,5282
C 19	141,0162
H 20	29,6803
H 21	29,4594
H 22	29,2623
H 23	28,6886
H 24	29,6379
H 25	29,7107
O 26	265,7563

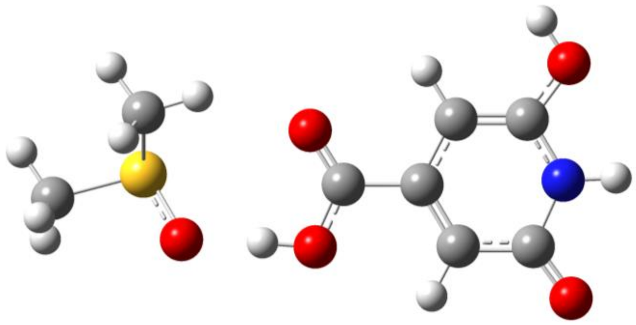

GIAO isotropic Magnetic Shielding (ppm) for CZA + DMSO system, with DMSO molecule near the hydroxyl group, calculated at the ωB97XD/6311++G(d,p)/PCM(dmso)//B3LYP/6311++G(d,p)/PCM(dmso)

C	1	21,248
C	2	95,3968
N	3	67,8963
O	4	201,1025
C	5	36,6728
C	6	12,12
O	7	-64,1792
O	8	130,6485
H	9	25,4269
H	10	26,6834
C	11	14,5089
H	12	18,2114
O	13	0,9035
C	14	65,6305
H	15	25,2465
H	16	25,9407
S	17	181,8258
C	18	141,4873
C	19	140,8071
H	20	29,6832
H	21	29,2578
H	22	29,494
H	23	29,6803
H	24	29,7337
H	25	28,2723
O	26	262,6697

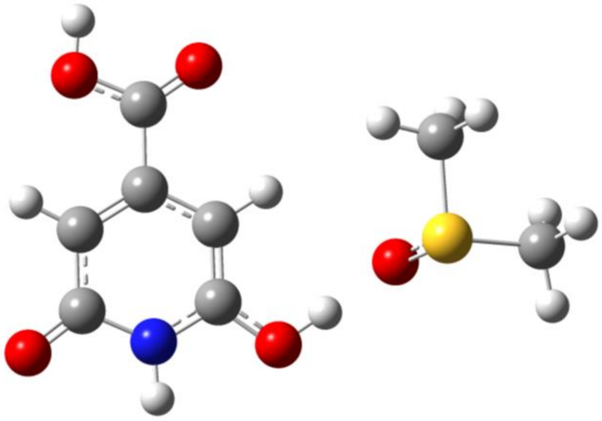

GIAO isotropic Magnetic Shielding (ppm) for CZA + DMSO system, with DMSO molecule near the NH group, calculated at the ωB97XD/6311++G(d,p)/PCM(dmso)//B3LYP/6311++G(d,p)/PCM(dmso)

C	1	19,238
C	2	94,5672
N	3	73,3525
O	4	174,9715
C	5	35,9487
C	6	11,8458
O	7	-64,2641
O	8	130,272
H	9	25,4633
H	10	18,4404
C	11	16,044
H	12	24,0162
O	13	5,8062
C	14	69,3658
H	15	25,3935
H	16	25,6217
S	17	180,5515
C	18	141,1065
C	19	141,506
H	20	29,6371
H	21	29,075
H	22	29,3627
H	23	29,3852
H	24	29,658
H	25	29,2278
O	26	259,378

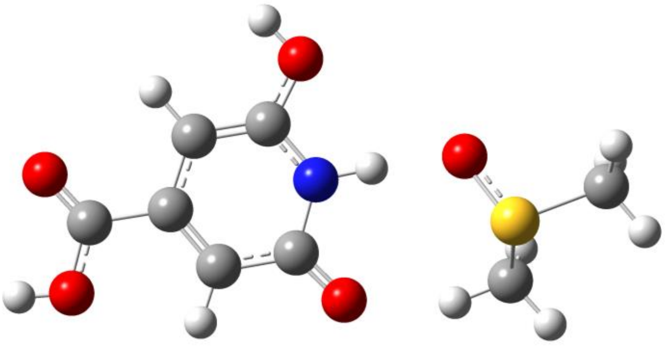

Supplement: Supplementary file 1 [file materials-14-00770-s001.pdf]
